# Dietary Inflammatory Index and the Risk of Gastric Precancerous Lesions Among Korean Adults in a Rural Area

**DOI:** 10.3390/nu17223502

**Published:** 2025-11-08

**Authors:** Yewon Cho, Dongkyu Lee, Chang Soo Eun, Dong Soo Han, Hyun Ja Kim

**Affiliations:** 1 Department of Food and Nutrition, Gangneung-Wonju National University, Gangneung 25457, Republic of Korea; staryewon1@naver.com (Y.C.); dklee@gwnu.ac.kr (D.L.); 2 Division of Gastroenterology, Department of Internal Medicine, Hanyang University Guri Hospital, 153 Gyeongchun-ro, Guri-si 11923, Republic of Korea; cseun@hanyang.ac.kr (C.S.E.); hands@hanyang.ac.kr (D.S.H.)

**Keywords:** gastric precancerous lesion, atrophic gastritis, intestinal metaplasia, dietary inflammatory index, *Helicobacter pylori* infection, Korean

## Abstract

**Background/Objectives**: Gastric cancer is known to occur through a multistep process from gastric precancerous lesions, such as atrophic gastritis, intestinal metaplasia, and gastric dysplasia. Gastric precancerous lesions may have different risk factors for each stage, and it can be prevented by an anti-inflammatory diet. In this study, we examined the association between the dietary inflammatory index (DII) and the risk of gastric precancerous lesions among adults in a rural area. Moreover, we analyzed the interaction between the DII and *H. pylori* infection in relation to the risk of gastric precancerous lesion. **Methods**: Among 711 participants who had a gastroscopy in a community cohort study, 564 subjects were included in this analysis and were divided into three groups (233 in normal, 128 in atrophic gastritis, and 203 in intestinal metaplasia). Atrophic gastritis and intestinal metaplasia were diagnosed by endoscopy and histopathology in accordance with the Updated Sydney System. DII was derived from a food-frequency questionnaire and categorized into tertiles. *H. pylori* infection was determined by the Campylobacter-like organism test. **Results**: *H. pylori* infection was significantly associated with the increased risk of intestinal metaplasia (OR = 2.75, 95% CI = 1.76–4.27), but not with atrophic gastritis. The inflammation diet itself was not associated with both the risk of atrophic gastritis (OR = 0.93, 95% CI = 0.53–1.64) and intestinal metaplasia (OR = 1.32, 95% CI = 0.78–2.24). However, the risk of intestinal metaplasia was more increased in the inflammatory diet group with *H. pylori* infection (OR = 3.35, 95% CI = 1.54–7.30) compared to the anti-inflammatory diet group without *H. pylori* infection. **Conclusions**: This study found that *H. pylori* infection increased the risk of intestinal metaplasia, and this risk was further enhanced by a pro-inflammatory diet, suggesting that both diet and infection management are important for prevention of gastric precancerous lesions.

## 1. Introduction

Gastric cancer is a commonly diagnosed cancer and the fourth-leading cause of cancer mortality in the Republic of Korea [1]. According to Lauren’s histological classification, gastric cancer is categorized into intestinal and diffuse types [2]. The intestinal type is known to develop through a multistep process, progressing from atrophic gastritis to intestinal metaplasia and subsequently to gastric dysplasia, which are collectively referred to as gastric precancerous lesions [3,4]. Atrophic gastritis is a histopathologic condition characterized by chronic inflammation of the gastric mucosa with progressive loss of native glandular cells [5,6], and intestinal metaplasia refers to the replacement of gastric epithelium with intestinal-type epithelium [5,6]. Among gastric precancerous lesions, gastric dysplasia (including polypoid lesions classified as gastric adenomas) has the highest malignant potential, with high-grade dysplasia particularly prone to progress to gastric adenocarcinoma [5,6]. Unlike the well-characterized adenoma–carcinoma sequence in colorectal cancer, gastric cancer has a heterogeneous progression and does not always develop from recognized precursor lesions. However, previous other studies have reported that atrophic gastritis and intestinal metaplasia increased the risk of gastric cancer by six-fold and ten-fold, respectively [7,8]. To reduce the economic burden and mortality attributable to gastric cancer, preventing the progression of gastric precancerous lesions is critical. Accordingly, identifying risk and protective factors for these lesions is essential.

Risk factors for gastric cancer and precancerous lesions include sex, age, dietary factors, smoking, alcohol drinking, family history of gastric cancer, and *Helicobacter pylori* (*H. pylori*) infection, and these factors are involved in combination rather than independently during gastric carcinogenesis [9,10,11]. In particular, *H. pylori* infection is a well-known risk factor for gastric precancerous lesions as well as gastric cancer, as it induces chronic inflammation and various pathological changes in the gastric mucosa [9].

Dietary factors for gastric precancerous lesions include a high-salt diet, pickled food, vitamin C, and vitamin A [12,13,14]. In addition, nutrients with anti-inflammatory properties have been associated with a lower risk of both gastric cancer [15] and gastric precancerous lesions [14,16,17,18]. In Japanese studies, intakes of yellow vegetables and beta-carotene showed a protective effect on atrophic gastritis [18], and vitamin A was a preventive factor for intestinal metaplasia [14]. In addition, sulfur compounds in garlic and omega-3 fatty acids are known to exert an anti-inflammatory reaction [16,17]. Although anti-inflammatory nutrients have the potential to control gastric inflammation in previous studies, diet is composed of various foods and nutrients and affects the development of disease in a complex manner. Therefore, it is necessary to assess its association with disease using a comprehensive dietary index, rather than focusing on a single anti-inflammatory food or nutrient. The dietary inflammatory index (DII) provides a composite measure of the inflammatory potential of the overall diet [19].

DII, developed by Shivappa et al. in 2014 [19], is a composite indicator to estimate the inflammatory potential of a diet, which was calculated by using the intake of a total of 45 nutrients and foods. In a case–control study on the association between gastric cancer and DII in an Italian population, inflammatory diets increased the risk of gastric cancer [20]. Similarly, a study performed in a Korean population also showed an increased risk of gastric cancer by inflammatory diets [21]. A recent meta-analysis including studies from Korea, China, and Japan, has reported that higher DII scores were associated with elevated inflammatory markers [22]. Furthermore, a recent population-based analysis using data from the Korean National Health and Nutrition Examination Survey (KNHANES) have reported that higher DII was linked to increased cancer mortality, providing direct evidence from a nationally representative Korean cohort [23,24].

*H. pylori* infection and diet-induced inflammation are both key contributors to gastric mucosal damage and may act synergistically in the carcinogenic process. This biological interaction is thought to occur through shared inflammatory pathways, including increased oxidative stress, activation of nuclear factor-κB (NF-κB), and cytokine-mediated immune responses, which collectively amplify mucosal inflammation and promote epithelial injury and metaplastic transformation [25]. However, to the best of our knowledge, no epidemiological study to date has specifically examined the association between the DII and gastric precancerous lesions, particularly in conjunction with *H. pylori* infection. This observation is based on a literature search of PubMed and Web of Science up to October 2025, which identified studies on DII and gastric cancer, but none addressing gastric precancerous lesions. Accordingly, there is a clear need to investigate the association between the DII and gastric precancerous lesions, focusing on potential interactions with *H. pylori* infection.

Therefore, this study aimed to examine the association between DII and gastric precancerous lesions among a rural Korean population, and to assess whether this association differed according to *H. pylori* infection.

## 2. Materials and Methods

### 2.1. Study Design and Participants

**Figure 1:**
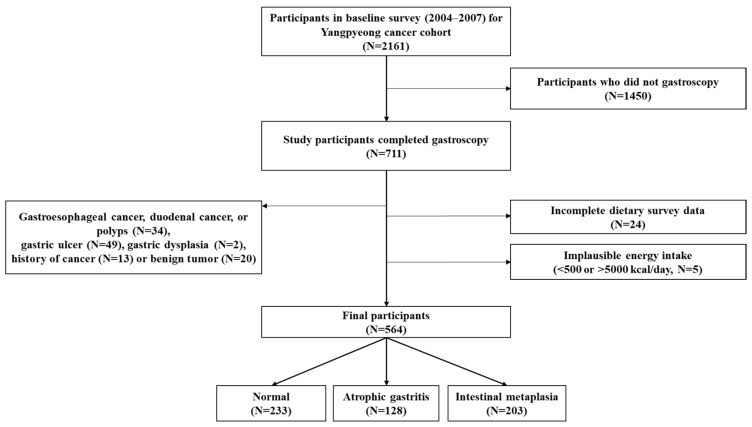
Flowchart of study subjects included in this study from the Yangpyeong cancer cohort.

This cross-sectional study used baseline data from a cancer cohort study in the Yangpyeong County. Among a total of 2161 adults who participated in the baseline survey between August 2003 and February 2007, 711 subjects voluntarily received gastroscopy. Of these, 564 subjects were finally included in the analysis after excluding individuals with the following conditions: gastroesophageal cancer, duodenal cancer and polyps (*n* = 34), gastric ulcer (*n* = 49), gastric dysplasia (*n* = 2), a history of cancer (*n* = 13) or benign tumor (*n* = 20), incomplete dietary survey data (*n* = 24), or implausible daily energy intake (<500 kcal or >5000 kcal; *n* = 5). Although gastric dysplasia is considered a gastric precancerous lesion, participants with this condition were excluded because their number was small (*n* = 2) and this lesion represents the final stage of the precancerous sequence, making it difficult to classify it within either the atrophic gastritis or intestinal metaplasia groups. The final 564 participants were classified into three groups: normal (*n* = 233), atrophic gastritis (*n* = 128), and intestinal metaplasia (*n* = 203) (Figure 1).

This study was approved by the Institutional Review Board of Hanyang University Hospital (IRB no. 2003–4), and written informed consent was obtained from all participants.

### 2.2. Gastroscopy and the Classification of Gastric Precancerous Lesions

Gastroscopy was performed by experienced physicians. For definitive diagnosis of gastric lesions, the stomach was divided into antrum, low-body, mid-body, and upper-body, and each of these regions were subdivided according to the direction (anterior wall, posterior wall, lesser curvature, and greater curvature), resulting in 16 predefined sites. Using one to four biopsies obtained from each antrum, low-body, mid-body, and upper-body of the stomach, an expert pathologist histologically confirmed the presence of gastric lesions and then classified them as normal mucosa, atrophic gastritis, or intestinal metaplasia according to the Updated Sydney System.

The final diagnosis of gastric lesions was made by considering both gastroscopic and histopathological results. Cases whose submucosal blood vessels were not observed in 16 sites of the stomach through a gastroscopic result were classified into the normal. Cases with one or more submucosal blood vessels were classified as the atrophic gastritis. Intestinal metaplasia was classified by the histopathological diagnosis. Cases with both atrophic gastritis and intestinal metaplasia were classified as the intestinal metaplasia.

To minimize potential interpretation errors due to different endoscopists over the long study period, three experienced gastroenterologists centrally re-reviewed 16 site-specific endoscopic photographs per participant in January 2008.

### 2.3. H. pylori Infection Test

The presence of *H. pylori* infection was examined by a rapid urease test using Campylobacter-like organism test (CLO; Kimberly Clark, Ballard Medical Products, Draper, UT, USA). Gastric mucosal biopsy specimens were placed into the urea-containing yellow gel, and a clear color change to orange or red within 24 h was interpreted as positive for *H. pylori.*

### 2.4. Questionnaire Survey

Height and weight of participants were measured while participants wore light gowns. The survey was performed by trained interviewers through direct face-to-face interviews. Questionnaire items consisted of socioeconomic factors (sex, age, education attainment, occupation, etc.), lifestyle habits (smoking, alcohol, and physical activity), medical history, family history of gastric cancer (first–degree relatives), and dietary factors (dietary habits and food-frequency information). A food-frequency questionnaire consisted of 117 food items, and intake frequency and a single amount for the past 1 year in the 3 years before interview were investigated. Intake frequency was divided into nine categories (none/less than once per month, 1–3 times per month, once per week, 2–4 times per week, 5–6 times per week, once per day, 2–3 times per day, 4–5 times per day, and ≥ 6 per day). Portion size was recorded as open-ended responses using easily recalled household units (e.g., bowls, dishes, piece, units) for each food item. All interviews and examinations were performed according to the standardized research protocol developed by the study team.

### 2.5. DII Measurement

DII was calculated using the method developed by Shivappa et al. [19]. Although 36 nutrients and 9 food items were included in the original method by Shivappa et al. [19], this study calculated DII including only 22 nutrients (7 inflammatory nutrients of energy, carbohydrate, protein, total fat, saturated fatty acids, cholesterol, and iron and 15 anti-inflammatory nutrients of mono- and poly-unsaturated fatty acids, omega-3 and -6 fatty acids, dietary fiber, folate, vitamin A, beta-carotene, thiamine, riboflavin, niacin, pyridoxine, zinc, vitamin C, and vitamin E) and 5 food items (garlic, ginger, onion, green or black tea, and pepper). Because 14 nutrients were not available in the nutrients database corresponding to the survey period, specifically the 7th Food Composition Table by the Korea Rural Development Administration and the Can-pro 3.0 nutrient database, and 4 food items (saffron, turmeric, thyme/oregano, and rosemary) were not commonly consumed in Korea, these were not included in the calculation of DII. In addition, we also excluded alcohol in DII items to control a potential multi-collinearity problem because alcohol consumption was adjusted in statistical models.

Z-scores for each nutrient or food item were calculated based on the global daily mean and standard deviation values reported by Shivappa et al. [19]. At this step, we used nutrient intakes adjusting for energy intake using the residual method [26]. To minimize a skewness of Z-distribution of each nutrients and food items, it was converted into a percentile score (0–1) and doubled, and followed by subtracting 1. And then, these values were multiplied by the overall inflammatory effect score of each nutrient or food item suggested by Shivappa et al. [19]; then, the DII of each participant was calculated by summing all the values. A negative DII value (<0) indicates an anti-inflammatory diet, while a positive value (≥0) indicates a pro-inflammatory diet.

### 2.6. Data Processing

Body mass index (BMI) was calculated as weight (kg)/height (m^2^), and categorized according to the World Health Organization (WHO) criteria for Asians underweight/ normal weight (≤22.9 kg/m^2^), overweight (≥23.0–24.9 kg/m^2^), or obese (≥25.0 kg/m^2^) [27]. Educational attainment was grouped into three categories (≤elementary school, middle school–high school, or ≥college). Smoking and drinking were each classified as never, past, or current. A family history of gastric cancer in first-degree relatives was divided into yes or no. *H. pylori* infection was classified into negative or positive. DII was categorized into tertiles based on its distribution in the normal group (Tertile 1, <−1.23; Tertile 2, −1.23–<0.95; Tertile 3, ≥0.95), with Tertile 1 as the reference category.

### 2.7. Statistical Analysis

General characteristics between the normal, atrophic gastritis, and intestinal metaplasia groups were compared using one-way ANOVA for continuous variables and the chi-square test for categorical variables. To examine the association between the DII and gastric precancerous lesions, we estimated odds ratios (OR) and 95% confidence intervals (95% CI) using multivariable logistic regression, adjusting for prespecified potential confounders (sex, age, BMI, education attainment, smoking, alcohol, *H. pylori* infection, and family history of gastric cancer). P for trend was analyzed by allocating the median value of their DII category to individuals and using these values as continuous variables in the logistic regression analysis. All statistical analyses were two-sided with α-error of 0.05 and performed using SAS software version 9.4 (SAS Institute., Cary, NC, USA).

## 3. Results

### 3.1. General Characteristics of the Study Participants

Table 1 presents the distribution of general characteristics and potential risk factors among the normal, atrophic gastritis, and intestinal metaplasia groups. The proportion of men was higher in the atrophic gastritis (39.8%) and intestinal metaplasia groups (45.8%) than in the normal group (30.5%) (*p* = 0.004). The mean age was higher in the atrophic gastritis (58.5 ± 10.9 years) and intestinal metaplasia groups (60.2 ± 9.7 years) than in the normal group (51.6 ± 12.1 years) (*p* < 0.001). The proportion of current smokers was higher in the intestinal metaplasia group (18.2%) than in the normal (11.2%) and atrophic gastritis groups (10.9%), but was not significant (*p* = 0.051). For *H. pylori* infection, the intestinal metaplasia group (73.0%) was higher compared to the normal (48.1%) and atrophic gastritis groups (56.3%) (*p* < 0.001). However, there was no significant difference between groups in BMI, education level, and daily mean energy intake.

### 3.2. Association Between Potential Risk Factors and the Risk of Gastric Precancerous Lesion

The risks of gastric precancerous lesions according to potential risk factors using multivariable logistic regression are presented in Table 2. The risks of atrophic gastritis (OR = 1.07, 95% CI = 1.04–1.09) and intestinal metaplasia (OR = 1.08, 95% CI = 1.05–1.11) compared to normal increased significantly with age. *H. pylori* infection was associated with a higher risk of intestinal metaplasia (OR = 2.75, 95% CI = 1.76–4.27), but not significant for atrophic gastritis (OR = 1.57, 95% CI = 0.98–2.51). Among the examined factors, age and *H. pylori* infection were the strongest predictors of intestinal metaplasia, whereas the DII did not show an independent association with either atrophic gastritis or intestinal metaplasia.

### 3.3. Association Between Dietary Inflammatory Index and the Risk of Gastric Precancerous Lesions

Table 3 presents the associations of the DII with gastric precancerous lesions. In both the age- and sex-adjusted model and the fully adjusted model (adjusting for age, sex, BMI, education attainment, smoking, drinking, *H. pylori* infection, and family history of gastric cancer), DII was not significantly associated with atrophic gastritis (OR = 0.93, 95% CI = 0.53–1.64) or intestinal metaplasia (OR = 1.32, 95% CI = 0.78–2.24).

### 3.4. Interaction of Dietary Inflammatory Index and H. pylori Infection in the Risk of Gastric Precancerous Lesion

The risks of gastric precancerous lesions according to *H. pylori* infection and DII were analyzed simultaneously and are presented in Table 4. After adjusting for confounding factors, the anti-inflammatory diet without *H. pylori* infection compared to the inflammatory diet with *H. pylori* infection was significantly associated with the risk of intestinal metaplasia (OR = 3.35, 95% CI = 1.54–7.30, interaction *p*-value < 0.01), but was not significantly associated with the risk of atrophic gastritis (OR = 1.61, 95% CI = 0.69–3.74).

## 4. Discussion

In rural Korean adults, H. pylori infection was significantly associated with intestinal metaplasia, while a higher DII was not. However, among the H. pylori-positive group, inflammatory diet was significantly associated with higher odds of intestinal metaplasia.

*H. pylori* infection is known to be a major risk factor for gastric precancerous lesions [9]. *H. pylori* infection causes chronic gastric inflammation which spreads to the gastric antrum or body leading to multiple atrophic gastritis and progresses to intestinal metaplasia [12]. In this study, there was no significant association between *H. pylori* infection and the risk of atrophic gastritis (OR = 1.57, 95% CI = 0.98–2.51). However, *H. pylori*-positive people had a 2.75-fold risk of intestinal metaplasia compared with *H. pylori*-negative people. This result suggests that *H. pylori* infection is a major risk factor of intestinal metaplasia but showed a difference from a previous report demonstrating a significant association with both atrophic gastritis and intestinal metaplasia [9]. *H. pylori* is primarily transmitted via person-to-person contact and typically acquired in early childhood [28,29,30]. In Korea, where the incidence of gastric cancer is high, the prevalence of *H. pylori* infection has gradually declined but still remains relatively high [31,32,33]. Previous studies have demonstrated that *H. pylori* eradication prevents the progression of gastric precancerous lesions and is particularly effective for intestinal-type gastric cancer [34,35,36,37,38]. Therefore, gastric precancerous lesions could be prevented by paying attention not to be infected by *H. pylori* in infancy or receiving eradication as presented in the previous study [29,34].

Several studies have reported that the DII was associated with the occurrence of a variety of diseases [20,21,39]. A recent multicenter case–control study in Brazil reported a positive association between pro-inflammatory diets and gastric adenocarcinoma, providing evidence that dietary inflammatory potential may contribute to gastric carcinogenesis [40]. In a case–control study analyzing the risk of colorectal cancer and DII, a pro-inflammatory diet increased the risk of colorectal cancer (OR = 2.16, 95% CI = 1.71–2.73) [39]. In addition, a pro-inflammatory diet significantly increased the risk of gastric cancer in a study performed in Korean (OR = 2.03, 95% CI = 1.09–3.77) [21] and Italian (OR = 2.35, 95% CI = 1.32–4.20) [20] populations. However, there has been no studies to analyze the relationship between the risk of gastric precancerous lesions and DII, although it has been reported that a high-salt diet and spicy foods increased the risk of gastric precancerous lesions [25,39,41]. In this study, DII showed no significant association with atrophic gastritis or intestinal metaplasia. This null association may be explained by the relatively narrow range of dietary inflammatory exposure within this population and the cross-sectional design, which limits the ability to infer temporal or cumulative effects of diet on early gastric mucosal changes. Similar limitations have been noted in other studies, where DII calculations based on a restricted number of food parameters or a limited exposure range might have attenuated associations with inflammation-related outcomes [42,43]. Intestinal-type gastric cancer develops through a multistep process from atrophic gastritis and intestinal metaplasia to gastric dysplasia [4], and it was reported that the risk factors for atrophic gastritis and intestinal metaplasia are different [44]. In this study, risk factors for atrophic gastritis was only old age. Risk factors for intestinal metaplasia included old age and *H. pylori* infection [44,45]. Therefore, it should be considered that risk factors for each stage of gastric precancerous lesions may be different. In addition, since the development process of gastric precancerous lesions to gastric cancer is not clear, further studies are needed to determine risk factors for gastric cancer in the presence of precancerous lesions.

In this study, with an anti-inflammatory diet, *H. pylori* infection significantly increased the risk of intestinal metaplasia (OR = 3.35, 95% CI = 1.54–7.30) compared to *H. pylori*-negative people. It has been suggested that gastric cancer as well as precancerous lesions can be prevented by attenuating *H. pylori* inflammation using several nutrients and foods and by applying eradication of *H. pylori* [46,47]. Sulfur-containing compounds in garlic and omega-3 fatty acids (e.g., EPA and DHA) are reported to exert an anti-inflammatory effect on *H. pylori*-induced inflammation [16,17]. The observed interaction between dietary inflammation and *H. pylori* infection may be explained by shared inflammatory pathways [48]. A pro-inflammatory diet could amplify oxidative stress and cytokine release induced by *H. pylori*, thereby accelerating mucosal injury and metaplastic changes [49]. This synergistic effect may involve enhanced activation of the NF-κB signaling pathway, increased production of reactive oxygen and nitrogen species, and suppression of gastric mucosal defense mechanisms, collectively promoting chronic inflammation and epithelial transformation. A recent population-based study has reported that higher DII scores were associated with increased systemic inflammatory markers, particularly among individuals with *H. pylori* infection [50]. These findings indicate that the coexistence of *H. pylori* infection and a pro-inflammatory diet may substantially increase the risk of intestinal metaplasia. These findings support our results, suggesting that dietary inflammatory potential may modify the effect of *H. pylori* on gastric mucosal transformation.

This study has several limitations. First, participants were rural adults and are less representative of Koreans. Nevertheless, recruitment encompassed all administrative areas of Yangpyeong-gun (1 eup and 11 myeons), which supports the representativeness of rural adults within the study region. In addition, when compared with the characteristics of a comparable age group of the 2005 KNHANES with a representative Korean population [51], the participants in Yangpyeong cohort showed a similar mean (± standard error) of energy intake (2016.3 ± 15.5 kcal/d in KNHANES vs. 2099.6 ± 44.2 kcal/d in Yangpyeong cohort) and BMI (24.4 ± 0.1 kg/m^2^ for men and 24.3 ± 0.1 kg/m^2^ for women in KNHANES vs. 24.8 ± 0.3 kg/m^2^ for men and 25.1 ± 0.3 kg/m^2^ for women in Yangpyeong cohort), and the level of educational attainment was also similar to that of rural populations in the national survey, reflecting typical characteristics of rural adults. Second, this study did not include all 45 DII components originally proposed by for Shivappa et al. [19]. Consistent with other Korean population-based studies, as several nutrients and foods could not be obtained from the national database or had insignificant intake levels, the DII was calculated using only the available items [21,23,52]. Moreover, a study calculating DII using 23 kinds of nutrients and foods in Korea verified the validity of this kind of research approach by demonstrating the association between serum high sensitivity C-reactive protein and DII [23]. Third, this study is a cross-sectional study using baseline data of the Yangpyeong community cancer cohort study, and it is thus difficult to explain the causality due to the unclear temporal order of exposure factors and results. However, since it is difficult to know the presence or absence of gastritis before gastroscopy, the possibility of diet control in the disease group is low, so it is very unlikely that the cause and effect have changed. Nevertheless, the findings should be interpreted as associations rather than evidence of causality, and further longitudinal studies are required to confirm these relationships. Fourth, the possibility of residual confounding from unmeasured factors, such as lifestyle behaviors or genetic susceptibility, cannot be entirely excluded. Despite these limitations, this study has several strengths. First, diet data of the study participants were collected using a food-frequency questionnaire. Because the food-frequency questionnaire investigates a routine food intake during a long period, it is a useful for analysis of the relationship between dietary intakes and disease, which is also suitable for this study using a long-term diet data. Second, the gastric lesions were clearly identified through gastroscopy. Gastroscopy was performed by gastroenterology physicians, and the diagnosis of gastric precancerous lesion was read by an expert pathology specialist using biopsy tissue. Moreover, to minimize reading errors, three expert gastroenterology physicians re-read the gastric precancerous lesions of all participants to further clarify. This rigorous endoscopic and histopathological protocol ensured high diagnostic accuracy and enhanced the reliability of the outcome classification in this study.

## 5. Conclusions

In this study, higher dietary inflammation index showed no significant association with gastric precancerous lesions overall. However, a significant association was observed among *H. pylori*-positive individuals. This suggests the potential importance of considering both dietary and infectious factors in gastric carcinogenesis process. In populations with a high prevalence of *H. pylori*, adopting anti-inflammatory dietary patterns, along with efforts to eradicate *H. pylori*, may contribute to the prevention of gastric precancerous lesions. Although causal inference is limited by the cross-sectional design, these findings provide preliminary evidence of a potential synergistic role between diet-induced inflammation and *H. pylori* infection in the early stages of gastric carcinogenesis. Future longitudinal or intervention studies are needed to confirm these associations and to clarify the underlying biological mechanisms.

## Figures and Tables

**Table 1:** General characteristics of the study participants.

	Normal (*n* = 233)	Atrophic Gastritis (*n* = 128)	Intestinal Metaplasia (*n* = 203)	*p*-Value ^(1)^
**Sex, *n* (%)**				
Men	70 (30.5)	5 (39.8)	93 (45.8)	0.004 *
Women	162 (69.5)	77 (60.2)	110 (54.2)	
**Age (years), mean ± SD**	51.6 ± 12.1 ^a^	58.5 ± 10.9 ^b^	60.2 ± 9.7 ^b^	<0.001 **
**Education, *n* (%)**				
≤Elementary school	108 (46.4)	69 (53.9)	119 (59.2)	0.086
Middle school to high school	107 (45.9)	53 (41.4)	73 (36.3)	
≥College	18 (7.7)	6 (4.7)	9 (4.5)	
**Smoking, *n* (%)**				
Non-smokers	177 (76.0)	92 (71.9)	129 (59.6)	0.051
Past smokers	30 (12.8)	22 (17.2)	37 (18.2)	
Current smokers	26 (11.2)	14 (10.9)	37 (18.2)	
**Drinking, *n* (%)**				
Non-drinkers	118 (50.6)	64 (50.0)	90 (44.4)	0.631
Past drinkers	10 (4.3)	4 (3.1)	11 (5.4)	
Current drinkers	105 (45.1)	60 (46.9)	102 (50.3)	
**BMI (kg/m^2^), *n* (%)**				
≤Normal weight (≤22.9)	65 (27.9)	41 (32.1)	65 (32.0)	0.754
Overweight (23.0–24.9)	60 (25.8)	32 (25.0)	56 (27.6)	
Obese (≥25)	108 (46.4)	55 (43.0)	82 (40.4)	
**Hypertension, *n* (%)**				
Normal	164 (70.4)	83 (64.8)	137 (67.5)	0.846
Present	63 (27.0)	42 (32.8)	61 (30.1)	
Unknown	6 (2.6)	3 (2.3)	5 (2.5)	
**Diabetes, *n* (%)**				0.002 *
Normal	214 (91.8)	117 (91.4)	178 (87.7)	
Present	13 (5.6)	8 (6.2)	19 (9.4)	
Unknown	6 (2.6)	3 (2.3)	6 (3.0)	
***H. pylori* infection, *n* (%)**				
Negative	120 (51.5)	56 (43.8)	64 (27.0)	<0.001 **
Positive	112 (48.1)	72 (56.3)	137 (73.0)	
**Family history of gastric cancer, *n* (%)**				
No	212 (91.0)	112 (88.2)	183 (90.2)	0.863
Yes	18 (7.7)	14 (11.0)	18 (8.9)	
**Energy intake(kcal/day), mean ±** **SD**	2092.2 ± 773.0	1926.4 ± 783.5	2012.1 ± 677.7	0.122

Abbreviations: SD, standard deviation; BMI, body mass index; *H. pylori*, *Helicobacter pylori*. ^(1)^ Statistical analysis was performed by one-way ANOVA for continuous variables and Pearson chi-square test for categorical variables. ^a,b^ Multiple comparison was performed by Duncan’s post hoc test. Different alphabet means a significant difference in means. * *p* < 0.01 ** *p* < 0.001

**Table 2:** Association between general risk factors and the risk of gastric precancerous lesion.

	Atrophic Gastritis (*n* = 128)			Intestinal Metaplasia (*n* = 203)		
	No. of Case/ Control	OR (95% CI) ^(1)^		No. of Case/ Control	OR (95% CI) ^(1)^	
**Sex**						
Men	51/71	1.00 (ref)		93/71	1.00 (ref)	
Women	77/162	0.52 (0.24–1.12)		110/162	0.30 (0.34–1.43)	
**Age (per 1 yr increase)**	128/233	1.07 (1.04–1.09) **		203/233	1.07 (1.05–1.11) **	
**BMI (kg/m^2^)**						
≤Normal weight (≤22.9)	41/65	1.00 (ref)		65/65	1.00 (ref)	
Overweight (23.0–24.9)	32/60	0.70 (0.38–1.31)		56/60	0.60 (0.46–1.45)	
Obese (≥25)	55/108	0.76 (0.44–1.30)		82/108	0.55 (0.48–1.33)	
**Education**						
≤Elementary school	69/108	1.00 (ref)		119/108	1.00 (ref)	
Middle school–High school	53/107	1.43 (0.80–2.54)		73/107	1.07 (0.73–2.06)	
≥College	6/18	0.67 (0.21–2.09)		9/18	0.50 (0.26–1.92)	
**Smoking**						
Non-smokers	92/177	1.00 (ref)		129/177	1.00 (ref)	
Past smokers	22/30	0.59 (0.25–1.42)		37/30	0.78 (0.39–1.97)	
Current smokers	14/26	0.71 (0.29–1.74)		37/26	1.21 (0.54–2.76)	
**Drinking**						
Non-drinkers	64/118	1.00 (ref)		90/118	1.00 (ref)	
Past drinkers	4/10	0.46 (0.12–1.72)		11/10	0.78 (0.28–2.16)	
Current drinkers	60/105	1.07 (0.62–1.86)		102/105	0.13 (0.68–1.88)	
***H. pylori* infection**						
Negative	56/120	1.00 (ref)		64/120	1.00 (ref)	
Positive	72/112	1.57 (0.98–2.51)		137/112	2.75 (1.76–4.27) **	
**Family history of gastric cancer**						
No	112/212	1.00 (ref)		183/212	1.00 (ref)	
Yes	14/18	1.27 (0.57–2.79)		18/18	1.03 (0.48–2.19)	

Abbreviations: OR, odds ratio; 95% CI, 95% confidence interval; BMI, body mass index; *H. pylori, Helicobacter pylori*. ^(1)^ calculated by multivariable logistic regression including sex, age, BMI, education, smoking status, drinking status, *H. pylori* infection, family history of gastric cancer. ** *p <* 0.001.

**Table 3:** Association between dietary inflammatory index and the risk of gastric precancerous lesion.

Gastric Precancerous Lesions	Dietary Inflammatory Index ^(2)^	No. of Case/Control	Age- and Sex-Adjusted OR (95% CI) ^(1)^	Multivariable-Adjusted OR (95% CI) ^(1)^
**Atrophic gastritis**				
	Tertile 1	45/77	1.00 (ref)	1.00 (ref)
	Tertile 2	38/78	0.88 (0.51–1.54)	0.89 (0.50–1.58)
	Tertile 3	45/78	0.93 (0.54–1.60)	0.93 (0.53–1.64)
	*p* for trend ^(3)^		0.780	0.803
**Intestinal metaplasia**				
	Tertile 1	60/77	1.00 (ref)	1.00 (ref)
	Tertile 2	69/78	1.21 (0.73–2.01)	1.18 (0.69–2.00)
	Tertile 3	74/78	1.23 (0.75–2.04)	1.32 (0.78–2.24)
	*p* for trend ^(3)^		0.114	0.303

Abbreviations: OR, odds ratio; 95% CI, 95% confidence interval. ^(1)^ Multivariable logistic regression adjusted for sex, age, BMI, education, *H. pylori* infection, smoking, drinking, and family history of gastric cancer. ^(2)^ Dietary inflammatory index were categorized into tertiles (Tertile 1: <−1.23, Tertile 2: −1.23 to 0.95, Tertile 3: ≥0.95) by dietary inflammatory index of normal group. ^(3)^ *p* for trends by multivariable logistic regression for continuous variables.

**Table 4:** Interaction of dietary inflammatory index and *H. pylori* infection on the risk of gastric precancerous lesion.

Gastric Precancerous Lesions		*H. pylori* Infection			
		Negative		Positive	
		No. of Case/ Control	Multivariable-Adjusted OR (95% CI) ^(1)^	No. of Case/ Control	Multivariable-Adjusted OR (95% CI) ^(1)^
**Atrophic gastritis**					
Dietary	Tertile 1	17/37	1.00 (ref)	28/40	1.45 (0.65–3.25)
inflammatory	Tertile 2	16/37	0.97 (0.41–2.29)	22/40	1.29 (0.57–2.94)
index ^(2)^	Tertile 3	23/46	0.81 (0.35–1.87)	22/32	1.61 (0.69–3.74)
**Intestinal metaplasia**					
Dietary	Tertile 1	22/37	1.00 (ref)	36/40	1.69 (0.78–3.65)
inflammatory	Tertile 2	18/37	1.87 (0.36–2.02)	51/40	2.79 (1.31–5.94) **
index ^(2)^	Tertile 3	24/46	0.88 (0.40–1.97)	50/32	3.35 (1.54–7.30) **

Abbreviations: OR, odds ratio; 95% CI, 95% confidence interval; *H. pylori, Helicobacter pylori*. ^(1)^ Multivariable logistic regression adjusted for age, sex, BMI, education, smoking, drinking, and family history of gastric cancer. ^(2)^ Dietary inflammatory index were categorized into tertiles (Tertile 1: <−1.23, Tertile 2: −1.23 to 0.95, Tertile 3: ≥0.95) by dietary inflammatory index of normal group. ** *p* < 0.01.

## Data Availability

The data presented in this study are available on request from the corresponding author. The data are not publicly available because of the privacy of the subjects.
